# Pregnancy and lactation-associated osteoporosis as a major type of premenopausal osteoporosis: a retrospective cohort study based on real-world data

**DOI:** 10.1186/s12884-024-06520-0

**Published:** 2024-04-22

**Authors:** Kyoko Kasahara, Sachiko Tanaka-Mizuno, Shunichiro Tsuji, Mizuki Ohashi, Makiko Kasahara, Taku Kawasaki, Takashi Murakami

**Affiliations:** 1 The Department of Obstetrics and Gynecology, Shiga University of Medical Science, Otsu, Shiga Japan; 2 The Laboratory of Epidemiology and Prevention, Kobe Pharmaceutical University, Kobe, Japan; 3 The Department of Orthopedic Surgery, Shiga University of Medical Science, Otsu, Shiga Japan

**Keywords:** Fracture, Lactation, Osteoporosis, Pregnancy, Premenopausal

## Abstract

**Background:**

Pregnancy and lactation-associated osteoporosis (PLO), as well as premenopausal osteoporosis, might be a predictor of future fracture. This study aimed to describe the clinical features of PLO as a subtype of premenopausal osteoporosis and to evaluate medical interventions for it.

**Methods:**

From an administrative claims database including 4,224,246 people in Japan, we classified women for whom the date of childbirth had been defined and who had suffered low-trauma fracture between the ages of 18–47 years as the premenopausal osteoporosis group. A fracture site for which the odds ratio for fractures occurring between 5 months before and 12 months after childbirth (around childbirth) was greater than 1 was considered the PLO site. We classified patients with a fracture at the PLO site around childbirth as the PLO group. The control group consisted of 500 women without fragility fractures. We investigated some drugs and diseases to explore fracture-causing factors, as well as medical interventions such as osteoporosis diagnosis, bone densitometry, anti-osteoporosis pharmacotherapy, and lactation inhibitors.

**Results:**

In total, 231 parous women were classified into the premenopausal osteoporosis group. The most common fracture was vertebral fracture and was likely to occur around childbirth, followed by distal radius and sacral fractures, which were rare around childbirth. Considering vertebral, pelvic, and proximal femoral fractures as PLO sites, 56 women with 57 PLO fractures were classified into the PLO group. The incidence of PLO was estimated at 460 per million deliveries. Ovulation disorder and high maternal age were associated with the development of PLO. Vertebral fracture was the most common PLO fracture. It was mainly diagnosed a few months, and possibly up to 1 year, postpartum. PLO patients with vertebral fractures underwent more medical interventions than did those with other fractures, but they were still inadequate.

**Conclusions:**

PLO with vertebral fracture was one of the major types of premenopausal osteoporosis. The prevalence of PLO is considered to be higher than previously thought, indicating the presence of potentially overlooked patients. More timely interventions for PLO might lead to the improved management of latent patients with premenopausal osteoporosis and reduce future fracture risk.

## Background

Osteoporotic fracture is a serious public health concern in aging societies. Especially in women, accelerated bone turnover after menopause is inevitable, so acquiring high bone mass in the late teen years and maintaining minimum bone loss throughout reproductive age are essential for reducing the risk of fragility fractures in old age. Previous studies have indicated that a premenopausal fracture would be a risk factor for subsequent fracture after menopause [1]. Women of reproductive age may experience conditions that adversely affect their bone metabolism, such as estrogen deficiency from various causes, diseases requiring glucocorticoids (GCs), poor nutrition, smoking, genetic factors, pregnancy, and lactation [2, 3]. Among these conditions, pregnancy and lactation are the most common. An improved understanding of the effects of these common life events is therefore important for improving both future fracture prevention and care for potential patients.

Pregnancy and lactation-associated osteoporosis (PLO) is a type of premenopausal osteoporosis in which pregnant and lactating women present with fragility fractures [3, 4]. Although pregnancy and lactation-related bone loss have previously been considered transient and reversible after breast milk weaning [5–8], a recent large study suggested that PLO could predict future fractures [9, 10], similar to premenopausal osteoporosis [1]. Moreover, long-term lactation might have an impact on decreased bone mass in the postmenopausal period [11].

PLO is such a rare and easily overlooked condition that its investigation requires well-designed large-scale studies. In addition, to date, no screening, diagnosis, or management strategies for PLO have been firmly established. Vertebral fractures during the late pregnancy and early postpartum periods have been the most typically described outcome of PLO, although other fractures are possible [9, 12, 13]. Nevertheless, a recent systematic review, as well as a recent social media survey of PLO, investigated only vertebral fractures [14, 15]. In Japan, a few epidemiological studies of osteoporosis in young women have recently been published [13, 16], but it is difficult to identify the occurrence of fragility fractures without major trauma in large populations with high anonymity. Toba et al. [13] investigated the epidemiology of PLO using the Japanese Diagnostic Procedure Combination (DPC) database covering about half of all inpatients in Japan, but single fragility fractures are often treated without hospitalization. The diagnosis of premenopausal osteoporosis including PLO should be considered in cases of fragility fracture without major trauma that occur as the result of falls from standing or lower positions, after various causes of pathological fracture have been ruled out. Moreover, bone mineral density (BMD) measurements alone should not be used to diagnose osteoporosis in premenopausal women because the relationship between BMD and fracture risk in that population differs from that in postmenopausal women [17, 18].

Given this background, the primary aim of this study was to describe the clinical features of PLO as a subtype of premenopausal osteoporosis. Secondarily, we evaluated medical interventions for women with PLO. To investigate this rare condition, we conducted epidemiological research using a health insurance database. Although we did not have the patients’ medical records or BMD values, the International Classification of Diseases, 10th revision (ICD-10) codes allowed us to distinguish low-trauma fractures from high-trauma and pathological fractures. Prior to our study of patients with PLO, we defined premenopausal osteoporosis as a low-trauma fracture in women of reproductive age involving the fragility fracture sites common among older people.

## Methods

### Data source

We conducted a retrospective cohort study using an administrative claims database maintained by the Japan Medical Data Center (JMDC, Tokyo, Japan). The JMDC database contains health insurance claims and ledger data from employee-based insurance systems from January 2005 to September 2017, including a total of 4,224,246 insured individuals in Japan. Disease diagnoses were recorded based on the ICD-10 codes (Table 1A). In the present study, the ICD-10 codes flagged for suspicion were regarded as invalid. All procedures were recorded by Japanese standardized procedure codes (Table 1B). Prescribed drugs were coded according to the Anatomical Therapeutic Chemical (ATC) Classification of both the European Pharmaceutical Market Research Association (EphMRA) and World Health Organization (WHO) (Table 1C). In addition, codes assigned to inpatients were discriminated from those assigned to outpatients by insurance type. The ledger data could define deliveries using the year and month of birth of the participant’s newborn if a mother had a baby in the insured periods and both were covered by the same insurance. However, to protect anonymity, our database did not provide the day of birth.

### Study participants

**Fig. 1:**
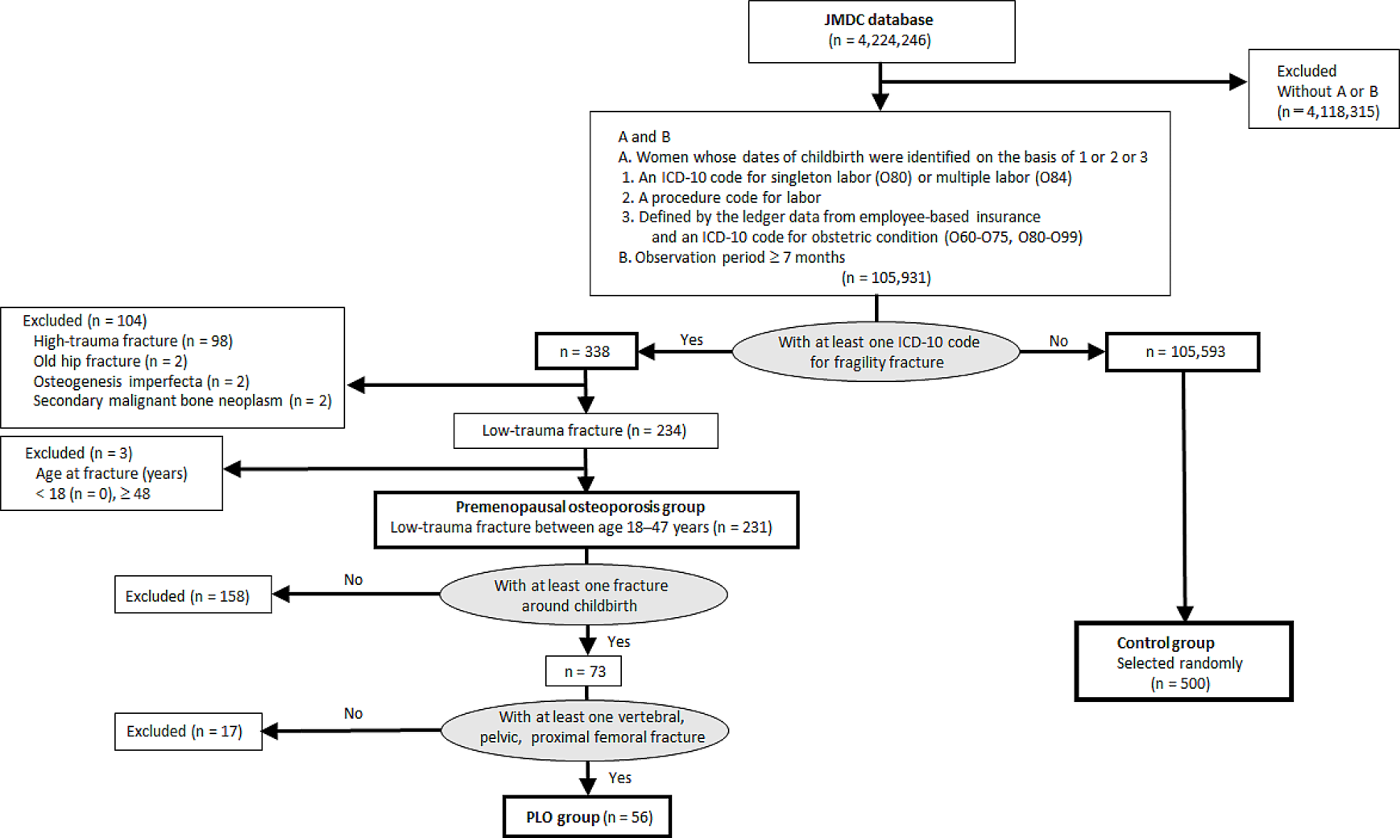
Flowchart of the study population Of 105,931 women for whom the date of childbirth was defined, 231 parous women who experienced a low-trauma fracture between the ages of 18–47 years were classified into the premenopausal osteoporosis group. Subsequently, 56 women were classified into the PLO group. The control group was created by randomly selecting 500 women without an ICD-10 code for fragility fracture. JMDC, the Japan Medical Data Center; ICD-10, International Classification of Diseases, 10th revision; PLO, pregnancy and lactation-associated osteoporosis

From the JMDC database, we extracted records of 105,931 women for whom the date of childbirth was defined (A) and who had been insured for at least 7 months (B) (Fig. 1). The inclusion criteria for (A) were as follows: A1, an ICD-10 code for singleton spontaneous delivery (O80) or multiple delivery (O84); A2, Japanese standardized procedure codes for obstetrical surgery or treatment (Table 1B); and A3, defined by the ledger data, as well as an ICD-10 code for obstetric conditions (O60-O75, O80-O99). Because deliveries defined by the ledger data could not exclude surrogate mothers or adopted children, the ICD-10 codes for obstetric conditions were required to ensure the mother’s delivery. The year and month of childbirth were considered to be those of the codes assigned (A1, A2), or those of the birth of the newborn (A3).

Next, we divided the records into two groups based on the presence or absence of at least one ICD-10 code for fracture: vertebral fractures including thoracic and lumbar fractures, sacral fractures, pubic fractures, pelvic fractures excluding pubic fractures, proximal humeral fractures, distal radius fractures, proximal femoral fractures, and sequelae of fractures such as old vertebral and old proximal femoral fractures (Table 1A).

The records of fractures were examined in detail for low-trauma fractures. Women with multiple fractures, transverse process fractures, dislocated fractures, and coincident severe traumatic conditions (e.g., spinal cord injury, traumatic intracranial hemorrhage, liver damage, kidney damage) were excluded as cases of high-trauma fracture. Old proximal femoral fractures that could be distinguished from new fractures by X-ray inspection were excluded. In contrast, old vertebral fractures were not excluded, since X-rays cannot precisely judge the freshness of a vertebral fracture. The women with other causes of pathological fracture, such as secondary malignant bone neoplasm or osteogenesis imperfecta, were also excluded. Subsequently, considering the average ages of menarche and menopause, women who experienced a fracture at ages outside the range of 18–47 years were excluded. We defined patients for whom the date of childbirth was defined and who had suffered a low-trauma fracture between the ages of 18–47 years as the premenopausal osteoporosis group. In this group, we investigated whether the ICD-10 codes for fracture had been assigned to inpatients or outpatients.

Next, to explore the impact of pregnancy and lactation on bone fragility, the temporal relationship between fracture and the nearest childbirth was investigated. Because the birthdates in our database were expressed on a monthly basis, the periods between fracture and childbirth were also expressed on a monthly basis. For example, fractures occurring in the previous, current, and next month of childbirth were classified as “–1 month”, “0 months”, and “+1 month”, respectively. Then, all fractures were classified into 18-month periods, between “–95 months and − 78 months”, “–77 months and − 60 months”, “–59 months and − 42 months”, “–41 months and − 24 months”, “–23 months and − 6 months”, “–5 months and + 12 months”, “+13 months and + 30 months”, “+31 months and + 48 months”, “+49 months and + 66 months”, “+67 months and + 84 months”, “+85 months and + 102 months”, and “+103 months and + 120 months”. In addition, the fractures classified into the period between “–5 months and + 12 months”, which could have occurred in association with pregnancy and lactation, were called “fractures around childbirth”. Moreover, to explore which fracture site was likely to be involved around childbirth, the odds ratio (OR) for “fractures around childbirth” was calculated by logistic regression for each fracture site. Then, considering fracture sites with such ORs greater than 1 as a PLO site, we defined fractures that occurred around childbirth at PLO sites as PLO fractures. In the premenopausal osteoporosis group, we defined patients who suffered at least one PLO fracture as the PLO group.

On the other hand, the control group was created by randomly selecting 500 of the women who met the inclusion criterion for (A) and (B) mentioned above, but had no code for fragility fracture, according to a computer-generated randomization list.

### Definition of variables

First, to explore fracture-causing factors, prescribed drugs and diseases that might cause bone fragility in reproductive age women were determined from the ATC (Table 1C) and ICD-10 codes (Table 1A), respectively. We regarded only ATC codes assigned before the first fracture in each case as valid in the PLO group.

Among the many drugs that can have adverse effects on bone health, we focused on GCs, antiseizure medications (ASMs), and anticoagulants. The short-term use of GCs, such as injections for fewer than 3 days and oral medications for fewer than 28 days, were excluded. ASMs related to osteoporosis in the practice guidelines for epilepsy in Japan (phenobarbital, primidone, phenytoin, carbamazepine, and valproic acid; Table 1C) were also investigated, excluding short-term use of fewer than 28 days. In addition, anticoagulants such as unfractionated heparin, low-molecular-weight heparin, and warfarin were investigated, whereas perioperative short-use heparin was excluded. After the prescription of these drugs was investigated, diseases requiring these drugs were explored.

In addition, the ICD-10 codes for rheumatoid arthritis (RA), diabetes mellitus (DM), epilepsy, thyroid dysfunction, and estrogen-deficient conditions, including eating disorders, “absent, scanty, and rare menstruation”, and “female infertility associated with anovulation”, were investigated. We also performed a search for “female infertility unspecified”.

Second, medical interventions for osteoporosis after a diagnosis of a fracture were investigated. The diagnosis of osteoporosis was determined from the ICD-10 codes (M80, M81). The examination for osteoporosis was determined from the procedure code for bone densitometry (D217). To investigate anti-osteoporosis pharmacotherapy, we searched for the ATC codes of the drugs physicians in Japan generally prescribe for osteoporosis, as follows: calcium, active vitamin D analogues, selective estrogen receptor modulators (SERMs), bisphosphonates, vitamin K, teriparatide, and denosumab (Table 1C). Under calcium, calcium gluconate hydrate (A12AA003) for systemic management was excluded. In addition, prolactin (PRL) secretion inhibitors at a therapeutic dose for lactation suppression, such as cabergoline, bromocriptine, and terguride, were also investigated.

### Statistical analysis

Comparative analysis was performed using Pearson’s chi-squared test for categorical variables and Student’s *t*-test for continuous variables. A two-sided significance level of 5% was used for all tests. All statistical analyses were performed using the SAS statistical software package (version 9.4; SAS Institute Inc., Cary, NC, USA).

## Results

Of 105,931 women who met our inclusion criteria, 338 had at least one ICD code for fragility fracture (Fig. 1). Of the 338 women with fragility fractures, 98 with high-trauma fractures, 2 with old proximal femoral fractures, 2 with secondary malignant bone neoplasms and primary cancer, and 2 with osteogenesis imperfecta were excluded. Next, of the 234 women with low-trauma fractures, 3 with fractures at age 48 years and over were excluded. No women developed low-trauma fractures at age 17 years or younger. Then, 231 parous women who experienced a low-trauma fracture between the ages of 18–47 years were classified into the premenopausal osteoporosis group. In this group, 237 fractures were found (four women had two fractures and one woman had three fractures). These 237 fractures were divided into 74 fractures around childbirth and 163 during other periods. The number of women who experienced at least one fracture around childbirth was 73, because one woman developed a pelvic fracture in the month before childbirth and a proximal femoral fracture 10 months after the same childbirth. In contrast, 158 women had no fracture around childbirth.

**Table 1:** List of codes investigated

**A**. ICD-10 codes	
**Fragility fracture**	**ICD-10 code starts with**
Vertebral fracture	
Thoracic fracture	S220, S221
Lumbar fracture	S320, S327
Fracture of spine, miscellaneous	T021, T08-
Sacral fracture	S321
Pubic fracture	S325
Pelvic fracture excluding pubic fracture	S328
Proximal humeral fracture	S422
Distal radius fracture	S525
Hip fracture	S720, S721, S722
Sequelae of fracture	
Old vertebral fracture	T911
Old hip fracture	T931
**Disease**	**ICD-10 code starts with**
Secondary malignant neoplasm of bone	C795
Idiopathic thrombocytopenic purpura	D693
Thyroid dysfunction	E00-E07
Diabetes mellitus	E10-E14
Addison’s disease	E271
Eating disorder	F50
Epilepsy	G40, G41
Respiratory diseases	J00-J99
Noninfective enteritis and colitis	K50-K52
Ulcerative colitis	K51
Rheumatoid arthritis	M069
Osteoporosis	M80, M81
Absent, scanty, and rare menstruation	N91
Female infertility associated with anovulation	N970
Female infertility unspecified	N979
Osteogenesis imperfecta	Q780
**B.** Japanese standardized procedure codes	
**Obstetrical surgery and treatment**	**Code**
Method of stopping uterine bleeding at the delivery	J077
Expulsion of placenta	J084
Kristeller maneuver	J085
Breech extraction	K892
Vacuum extraction	K893
Forceps delivery	K894
Episiotomy and suture	K895
Suture of perineal laceration	K896
Suture of cervical laceration	K897
Cesarean section	K898
Bimanual compression of the uterus	K901
Manual removal of placenta	K902
Vaginal manipulation of uterine inversion	K905
**C.** ATC codes	
**Drug**	**EphMRA code**
Unfractionated heparin	B01B1
Low molecular weight heparin	B01B2
Glucocorticoid for injection	H02A1
Glucocorticoid for oral administration	H02A2
Drug for osteoporosis	
Calcium	A12A
Active vitamin D analogues	A11C2
Selective estrogen receptor modulators	G03J
Bisphosphonates	M05B3
**Drug**	**WHO code**
Anticoagulant	
Warfarin	B01AA03
Antiseizure medications related to osteoporosis	
Phenobarbital	N03AA02
Primidone	N03AA03
Phenytoin	N03AB02
Carbamazepine	N03AF01
Valproic acid	N03AG01
Drug for osteoporosis	
Vitamin K	B02BA
Teriparatide	H05AA02
Denosumab	M05BX04
Drug for lactation suppression	
Terguride	G02CB06
Bromocriptine	N04BC01
Cabergoline	N04BC06

ICD-10, International Classification of Diseases, 10th revision

ATC, Anatomical Therapeutic Chemical Classification System;

EphMRA, European Pharmaceutical Market Research Association;

WHO, World Health Organization

**Table 2:** Fracture sites in the premenopausal osteoporosis group compared by period

	Total	During the 18 months around childbirth	Other periods	OR	95%CI
Number of fractures	237	74	163		
Fracture age (years), mean (SD)	34.6 (6.0)	34.1 (4.9)	34.8 (6.5)		
Fracture site					
Vertebrae	83	**45**	38	5.10	2.83–9.92
Pubis	7	**6**	1	14.30	1.69–121.0
Pelvis excluding pubis	7	**4**	3	3.05	0.66–14.0
Proximal femur	4	**2**	2	2.24	0.31–16.20
Proximal humerus	18	4	14	0.61	0.19–1.91
Sacrum	50	8	42	0.35	0.16–0.79
Distal radius	68	5	63	0.12	0.044–0.30

Number, odds ratios (ORs), and 95% confidence intervals (CIs) were calculated by logistic regression. Numbers in bold indicate pregnancy and lactation-associated osteoporosis fractures

A comparison of fracture sites around childbirth and those during other periods in the premenopausal osteoporosis group is shown in Table 2. No significant difference was seen in age at fracture between the two groups (*P* = 0.879). In the premenopausal osteoporosis group, the most common fracture site was the vertebrae (83/237 [35.0%]), followed by the distal radius and sacrum (68/237 [28.7%] and 50/237 [21.1%], respectively). Proximal femoral fracture, a major fragility fracture among older women, was rare (4/237 [1.7%]).

**Fig. 2:**
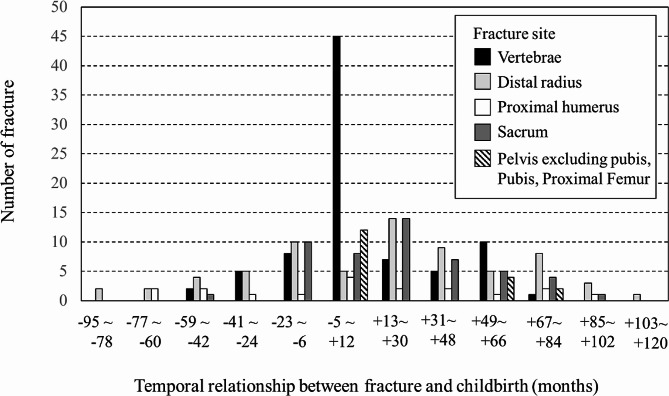
Temporal distribution of the low-trauma fractures in the premenopausal osteoporosis group The fracture onsets are presented separately by 18-month period. Vertebral fractures were the majority of fractures around childbirth (between 5 months before and 12 months after childbirth); however, they were not as frequent in other periods. Conversely, the frequencies of distal radius and sacral fractures were lower around childbirth than in other periods, during which they were common

The temporal distributions of a total of 237 low-trauma fractures in the premenopausal osteoporosis group are shown by period between fracture and childbirth in Fig. 2. Vertebral fractures, distal radius fractures, proximal humeral fractures, sacral fractures, and other fractures (pelvic fractures excluding pubic fractures, pubic fractures, and proximal femoral fractures) are presented separately by 18-month periods. The highest number of fractures, including all types, (*n* = 74) occurred around childbirth; they occurred far less frequently in other periods. Conversely, the frequencies of distal radius and sacral fractures were lower around childbirth than during other periods wherein they were common.

Next, vertebral fractures, pubic fractures, pelvic fractures excluding pubic fractures, and proximal femoral fractures, of which the ORs for “fractures around childbirth” were greater than 1 (Table 2), were considered PLO sites. Though the ORs for the latter two fractures were not significant, they were included as PLO sites because their small numbers might have explained the lack of significance. Of the 231 patients with premenopausal osteoporosis, 158 without fractures around childbirth were excluded (Fig. 1). Of the 73 patients with at least one fracture around childbirth, 17 without PLO fractures (i.e., those who had only distal radius, sacral, or proximal humeral fracture) were excluded. Finally, 56 PLO patients with 57 PLO fractures were identified as the PLO group. In this group, vertebral fractures were the most common (78.9% [45/57]), followed by pelvic fractures (17.5% [10/57]) and proximal femoral fractures (3.5% [2/57]) (Table 2). In contrast, the control group was created by randomly selecting 500 of 105,593 women without a code for fragility fracture.

**Table 3:** Baseline characteristics of the pregnancy and lactation-associated osteoporosis (PLO) and control groups

	PLO	Control	*P* value
No. of subjects	56	500	
No. of deliveries	65	581	
1, n (%)	47 (83.9)	424 (84.8)	0.845
2, n (%)	9 (16.1)	71 (14.2)	0.689
3, n (%)	0	5 (1.0)	1
Maternal age (years), mean (SD)	34.1 (5.2)	32.3 (4.8)	0.0082*
Maternal age (years), range	21.0–43.1	18.3–45.1	

SD, standard deviation. *P < 0.01

### Comparison of the PLO and control groups

A comparison of the baseline characteristics of the two study groups is shown in Table 3. The 56 patients in the PLO group delivered 65 singleton babies, whereas the 500 women in the control group delivered 581 singleton babies. Compared with the control group, maternal age was significantly higher in the PLO group (*P* = 0.0082).

The number of deliveries per woman in the PLO group (1.161) was equivalent to that in the control group (1.162). Based on these numbers, we considered that the number of deliveries among the 105,931 women who formed our study group was about 123,000, suggesting that the incidence of PLO would be about 460 per million deliveries.

**Table 4:** Comparison of fracture-causing factors in the pregnancy and lactation-associated osteoporosis (PLO) and control groups

	PLO (*n* = 56)	Control (*n* = 500)	OR	95%CI
Prescribed drugs				
Glucocorticoids	3 (5.4%)	20 (4.2%)	1.25	0.36–4.40
Antiseizure medications	1 (1.8%)	2 (0.4%)	4.13	0.37–46.50
Disease				
Rheumatoid arthritis	1 (1.8%)	5 (1.0%)	1.69	0.19–15.30
Diabetes mellitus	5 (8.9%)	38 (10.3%)	0.97	0.36–2.60
Eating disorder	1 (1.8%)	2 (0.4%)	4.33	0.38–48.8
Epilepsy	1 (1.8%)	5 (1.0%)	1.44	0.16–12.7
Absent, scanty, or rare menstruation	6 (10.7%)	14 (2.8%)	3.80	1.38–10.5
Female infertility associated with anovulation	17 (30.4%)	79 (15.8%)	2.10	1.12–3.92
Female infertility unspecified	20 (35.7%)	98 (19.6%)	2.02	1.11–3.68

Number, age-adjusted odds ratios (ORs), and 95% confidence intervals (CIs) were calculated by logistic regression

A comparison of fracture-causing factors between the PLO and control groups is shown in Table 4. ORs were estimated using logistic regression, which included the maternal age for deliveries complicated with PLO fractures in the PLO group and that for the latest delivery in the control group as an adjusted variable. Compared with the control group, “absent, scanty, and rare menstruation”, “female infertility associated with anovulation”, and “female infertility unspecified” were significantly more frequent in the PLO group (OR = 3.80, 95% CI: 1.38–10.5; OR = 2.10, 95% CI: 1.12–3.92; and OR = 2.02, 95% CI: 1.11–3.68, respectively). No significant differences in the frequency of other factors were seen between the two groups. The details of the diseases treated with GCs were as follows: respiratory diseases 16 (2 PLO and 14 controls), dermatosis 4 (4 controls), and autoimmune diseases 3 (1 case of ulcerative colitis in PLO, 1 case of Addison’s disease, and 1 case of idiopathic thrombocytopenic purpura in the controls). No patient with RA in either group was given GCs. All ASM users had epilepsy, but none had bipolar disorder.

The use of anticoagulants was so rare that we did not analyze their effects. In the control group, two patients with antiphospholipid syndrome were given heparin calcium during pregnancy, but no warfarin use was found. In the PLO group, one patient was given heparin calcium during pregnancy and warfarin after childbirth to treat thrombosis. The use of low-molecular-weight heparin injections was not found in either group. In addition, the effect of hyperthyroidism and hypothyroidism was not examined statistically, because there were only a few patients with these conditions, and some of them had codes for “Absent, scanty, or rare menstruation”, “Female infertility associated with anovulation”, or “Female infertility unspecified” concurrently.

### Temporal distributions of PLO fractures

**Fig. 3:**
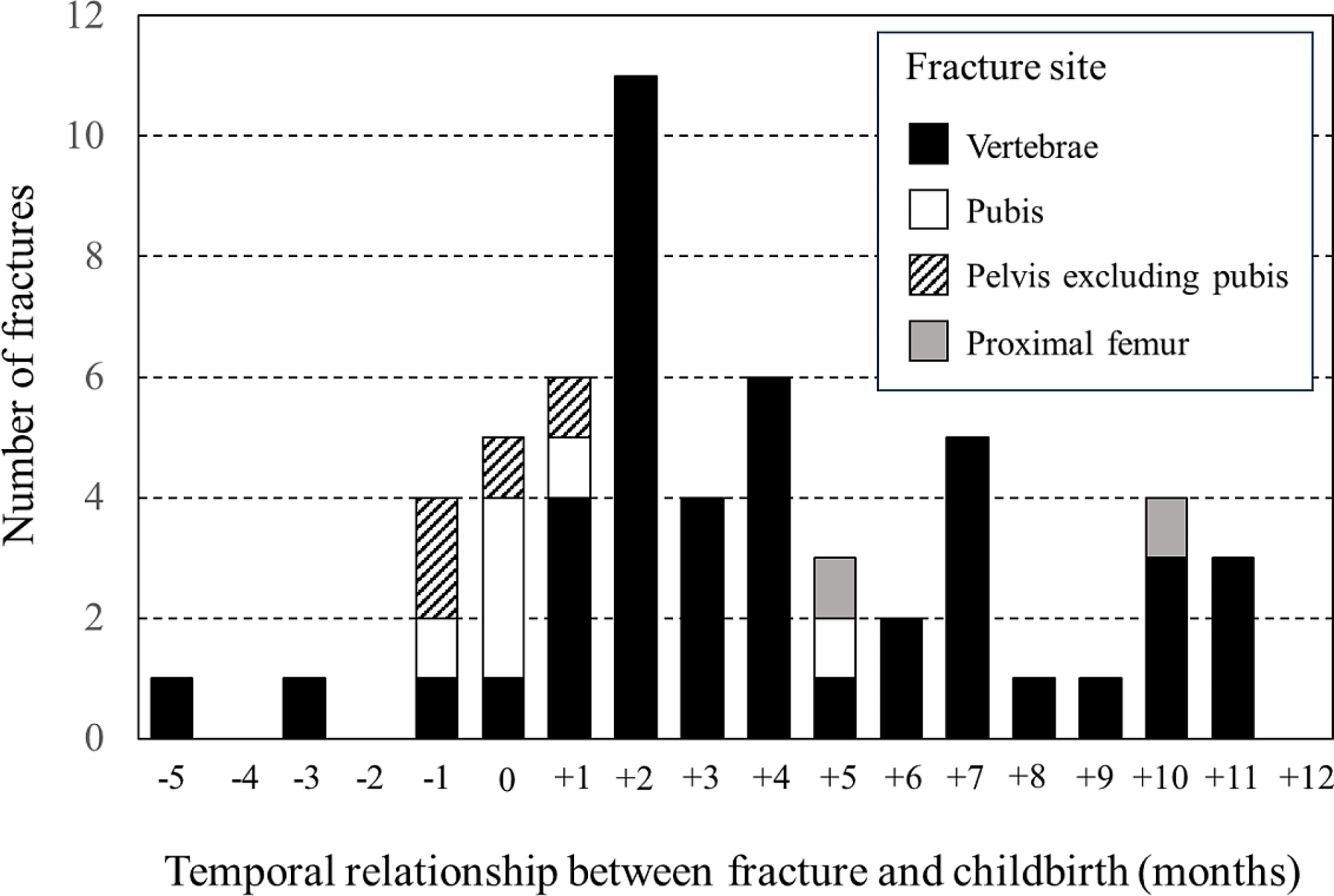
Temporal distribution of the low-trauma fractures in the pregnancy and lactation-associated osteoporosis group The fracture onsets are presented monthly by temporal relationship with childbirth. Vertebral fractures formed a peak at 2 months after childbirth, whereas pelvic fractures including pubic fractures occurred mostly between 1 month before and 1 month after childbirth

The temporal distributions of a total of 74 PLO fractures in the PLO group are shown by period between fracture and childbirth in Fig. 3. The numbers of fractures that occurred before the month of childbirth, the same month of childbirth, and after the month of childbirth were 6, 5, and 46, respectively. The occurrence of vertebral fractures formed a peak at 2 months after childbirth and gradually decreased at about 1 year after childbirth. On the other hand, most pubic fractures (5/6) and all pelvic fractures excluding pubic fractures (4/4) occurred between the month before and the month after childbirth. Considering both the former and latter, pelvic fractures were most likely to occur in the month of childbirth.

### Medical interventions for premenopausal osteoporosis including PLO

Of the 231 patients in the premenopausal osteoporosis group, 34 (14.7%) were diagnosed as having osteoporosis, 32 (13.9%) underwent bone densitometry, 25 (10.8%) were given anti-osteoporosis pharmacotherapy, and 10 (4.3%) were given lactation inhibitors. Of the 81 patients with vertebral fractures in this group, 31 (38.3%) were diagnosed as having osteoporosis, 28 (34.6%) underwent bone densitometry, 23 (28.4%) were given anti-osteoporosis pharmacotherapy, and 8 (9.9%) were given lactation inhibitors. In addition, these medical interventions were significantly more frequent in cases with compared to those without vertebral fracture (ORs = 30.40, 19.30, 29.30, and 8.11; 95%CIs: 8.90–104.0, 6.46–57.60, 6.70–128.0, and 1.68–39.2, respectively). On the other hand, of the 73 patients with fractures around childbirth, 26 (35.6%) were diagnosed as having osteoporosis, 22 (30.1%) underwent bone densitometry, 20 (27.4%) were given anti-osteoporosis pharmacotherapy, and 6 (8.2%) were given lactation inhibitors. These 73 patients showed a significantly higher frequency in the former three interventions than did the other 158 patients (ORs = 10.40, 6.38, and 11.50; 95%CIs: 4.40–24.50, 2.83–14.40, and 4.13–32.30, respectively); however, no difference in lactation inhibitors was found (OR = 3.45, 95%CI: 0.94–12.60).

**Table 5:** Medical interventions among patients with pregnancy and lactation-associated osteoporosis with vertebral fractures

	Total	Temporal relationship between fracture and childbirth (months)		
	Number (%)	-5 months – 0 months Number (%)	+ 1 month – +6 months Number (%)	+ 7 months – +12 months Number (%)
Patients	45 (100)	4 (100)	28 (100)	13 (100)
Diagnosis of osteoporosis	26 (57.8)	2 (50.0)	19 (67.9)	5 (38.5)
Bone densitometry	22 (48.9)	2 (50.0)	16 (57.1)	4(30.8)
Drug for osteoporosis	20 (44.4)	1 (25.0)	14 (50.0)	5 (38.5)
Drug for lactation suppression	6 (13.3)	1(25.0)	5 (17.9)	0

Next, the investigation of medical interventions for the PLO group (*n* = 56) showed that 11 patients without vertebral fractures did not receive any interventions. The number of patients with vertebral fractures who underwent a medical intervention is shown by period between fracture and childbirth in Table 5. The frequency of these interventions was highest 1–6 months after childbirth, except for lactation inhibitors. Lactation inhibitors were given to one patient with a fracture in pregnancy at the month of childbirth, and to 5 patients with fractures following childbirth in the month that they experienced the fracture or the month after that (13.3% [6/45]). All patients given lactation inhibitors were also treated with anti-osteoporosis drugs.

**Table 6:** Anti-osteoporosis pharmacotherapy for vertebral fracture in the PLO group

	Patients given drug therapy for osteoporosis (*n* = 20)
Active vitamin D analogues, bisphosphonates	3
Active vitamin D analogues, calcium	3
Active vitamin D analogues, vitamin K	2
Active vitamin D analogues, denosumab	2
Active vitamin D analogues	2
Bisphosphonates	4
Calcium	3
Teriparatide	1

In the PLO group, 20 of 45 patients with vertebral fracture were given various types of anti-osteoporosis pharmacotherapy (Table 6). Active vitamin D analogues were the most frequent drugs (*n* = 12), followed by bisphosphonates (*n* = 7) and calcium (*n* = 6). Half of the drug-treated cases (*n* = 10) were given a combination of active vitamin D analogues and another drug, 75% (*n* = 15) were given vitamin D or calcium, and teriparatide was prescribed to only one patient. SERMs were not used.

### Hospitalization rates among patients in the premenopausal osteoporosis and PLO groups

In the premenopausal osteoporosis group, 35 fractures required hospitalization (10 vertebral fractures, 5 pubic fractures, 2 pelvic fractures excluding pubic fractures, 3 proximal femoral fractures, 2 proximal humeral fractures, and 13 distal radius fractures). The total hospitalization rate was 14.8% (35/237), and this rate was especially high for proximal femoral fractures (75% [3/4]). In the PLO group, 8 PLO fractures required hospitalization (5 vertebral fractures, 2 pelvic fractures excluding pubic fractures, and 1 proximal femoral fracture). All five pubic fractures were diagnosed during hospitalization due to delivery.

## Discussion

Though it is widely accepted that childbirth, which is a major life event for the majority of women, is a potential risk factor for fragility fractures among premenopausal women, new epidemiological evidence supporting this are presented. Using real-world data from a total of 105,931 parous women, 56 women with a high probability of developing PLO were identified with the limitations of a database study.

Among the 237 fractures in the premenopausal osteoporosis group, the most common fracture site was the vertebrae, followed by the distal radius and sacrum (Table 2). These results do not contradict previous studies indicating that the incidence of vertebral and wrist fractures begins to increase rapidly soon after menopause [19], or those reporting a higher occurrence of distal radius compared with other fragility fractures in relatively younger people [20]. In contrast, the incidence of proximal femoral fractures was low compared with that in older people [21]. This is thought to be because younger people with good reflexes can still break their fall with their hands if they stumble, and thus they rarely land on their hip.

In the premenopausal osteoporosis group, 31% (74/237) of the low-trauma fractures occurred in the 18 months around childbirth, and 60% (45/74) of such fractures involved the vertebrae (Table 2). Meanwhile, 54% (45/83) of the vertebral fractures, which were the most frequent in premenopausal osteoporosis, developed around childbirth. Because we defined our study population as women for whom the date of childbirth was known, they tended to have ICD-10 codes for various conditions assigned close to their date of childbirth. Thus, the true incidence of premenopausal osteoporosis in the 18 months around childbirth should be lower than that shown in this study. Nevertheless, we believe our results demonstrate that vertebral fracture around childbirth is one of the major types of premenopausal osteoporosis.

In addition, we found that distal radius and sacral fractures, fragility fractures at cortical sites, were less frequent around childbirth; however, they were rather common in other periods at reproductive age (Table 2; Fig. 2). Bone turnover resulting from hormonal changes due to pregnancy and lactation would typically be related to trabecular bone loss rather than a thin cortex. In addition, pregnancy generally leads to increased body weight and excessive lumbar lordosis in women [3]. The spine might be vulnerable to such weight and postural loads, both of which can continue throughout the childcare period. Moreover, when pregnant or lactating women stumble and lose their balance, they are more likely to fall backward rather than forward as their instinct is to protect their abdomen or a baby in their arms. Thus, vertebral fractures tend to occur around childbirth, whereas distal radius fractures are rare, because they are usually caused by falling forward. In contrast, sacral fractures, which are often caused by falling on one’s buttocks, were rare around childbirth. The fracture type related to falling on one’s backside might depend on the presence or absence of adverse effects of pregnancy and lactation on the spine.

Furthermore, we defined vertebral fractures, pelvic fractures including pubic fractures, and proximal femoral fractures occurring in the 18 months around childbirth as PLO fractures. In the PLO group, vertebral fractures were the most common (78.9% [45/57]). The details of PLO fracture sites in this study differed from those in an analysis of DPC data covering only inpatients [13]. In our study, the total hospitalization rate in the premenopausal osteoporosis group was 14.8%, which was relatively high for proximal femoral fractures (*n* = 4) compared with that for other fractures. In practice, a single fragility fracture does not necessarily require hospitalization, except for proximal femoral fractures, which typically require surgery. Besides, we carefully selected low-trauma fractures to define the premenopausal osteoporosis group by excluding multiple fractures; in contrast, the DPC study included many multiple fractures. In addition, the PLO sites in our study were weight-bearing joints, sustaining increased maternal and offspring weight, which would thus be vulnerable during the late pregnancy and postpartum child-rearing periods. Therefore, we believe that our results are more accurate than those reported by the prior study.

In the present study, the incidence of PLO was estimated to be 460 per million childbirths, which was greater than the previous reported incidence of 4–8 per million pregnancies [22]; however, this incidence was similar to the results of an analysis of the DPC database [13], although that study population was different from the present one in various ways. What that study had in common with the present one, however, was that it also epidemiologically investigated fractures after the early postpartum period, although a diagnostic criterion has not yet been established for PLO. The present results suggest that there are potentially overlooked or undertreated patients who are otherwise healthy.

The present results showed that PLO fractures were clearly more frequent after than before childbirth (Fig. 3). In the PLO group, the frequency of vertebral fractures was highest 2 months after childbirth, and approximately half occurred later than 3 months after childbirth. These findings differ from those of previous case series claiming that PLO fractures typically occur in the third trimester of pregnancy and early postpartum period [12, 22]. Our results were similar to those of a recent systematic review showing that PLO occurs most frequently in the first 3 months after childbirth [14], but different with regard to the frequency later than 3 months after childbirth, which was 51.1% (24/47) in our study and 4.9% (4/82) in theirs. A very recent survey of PLO showed that more than one-fifth of vertebral fractures occurred later than 12 weeks after childbirth [15]. One explanation is that a systematic review investigating case reports and series would easily miss underlying patients in whom the physicians had no interest. Nevertheless, in clinical practice, the diagnosis of vertebral fractures is often delayed. Moreover, X-ray examinations are often avoided during pregnancy. Though the incidence of PLO was greater than that previously reported, it is difficult to state that fractures actually occur most commonly 2 months after childbirth and are frequent even more than 3 months after childbirth.

The temporal distribution of PLO fractures in our study was rather consistent with studies on BMD titers and biochemical markers of bone turnover in lactating women [5, 23]. Pregnancy and lactation are well known to be conditions involving high bone turnover to provide offspring with sufficient calcium for growth. Although a physiological increase in intestinal calcium absorption can protect maternal bone mass during pregnancy [24], the maternal skeleton undergoes resorption in women with low calcium intake. In addition, variations in the sensitivity to parathyroid hormone-related protein (PTHrP) released by the breasts and placenta may modulate the magnitude of bone resorption in normal pregnancy [3]. After childbirth, the calcium requirements of offspring increase and the enhanced intestinal calcium absorption disappears. Most of the calcium content in breast milk is provided from maternal skeletal resorption, which is mainly stimulated by high PTHrP and low estrogen levels. Hypoestrogenism in the early postpartum period is associated with placental expulsion, as well as elevated levels of PRL due to lactation. PRL inhibits the secretion of gonadotropin-releasing hormone and also directly interferes with ovarian follicular development.

Some studies have shown that bone loss after childbirth is related to the duration of lactation, as well as amenorrhea. It has been reported that 3–6 months of lactation can cause a 3–8% reduction in spine and hip BMD [6, 7], which is restored 6–12 months after weaning, whereas lactation for more than 9 months can delay such BMD recovery [25, 26]. Other reports have found bone loss due to lactation continuing for 18 months or longer, suggesting that the resumption of menstruation could be a main predictor of bone recovery [5, 23]. During lactation, the resumption of ovarian function depends on the suckling intensity of infants. Even fully lactating women restart ovulation at 10 weeks postpartum [27], and recover completely by 72 weeks after childbirth [28].

Our finding that ovulation disorder was highly associated with the occurrence of PLO was predictable (Table 4). It is not surprising that women in a hypoestrogenic state are more likely to experience fragility fractures, especially when they have to provide their offspring with sufficient calcium. Further, women who become pregnant through ovulation induction might need a long time to restart ovulation without such treatment, regardless of whether they lactate. We presume that the temporal distribution of PLO shown in Fig. 3 might be related to not only high bone turnover due to lactation, but also the delayed resumption of ovarian function after childbirth [7, 26].

A history of infertility treatment may also be associated with PLO (Table 4) through high maternal age (Table 3). Some authors have shown that PLO is an age-related disease [9, 13, 14]. As is well known, in women, a natural decrease in BMD, as well as a natural increase in fracture incidence, starts from around the midpoint of reproductive age and then proceeds gradually toward menopause [29]. Moreover, maternal age might be positively associated with increased bone turnover during lactation and the postweaning period [7]. Therefore, the increasing maternal age in developed countries might be associated with an increased risk of PLO.

In contrast, the drugs and conditions studied, except for ovulation disorder and infertility, rarely caused PLO (Table 4). We suspect that few patients with serious autoimmune diseases, epilepsy, or DM were included in our study group, which consisted of parous women of reproductive age. Generally speaking, physicians do not like to give medicine to female patients with mild disease who wish to have children. Moreover, patients with mild disease rarely have poor bone health in the pre-pregnancy period.

Unfortunately, we found that most cases of premenopausal osteoporosis were not treated as osteoporosis. The low rate of medication use following fragility fracture has often been reported in postmenopausal women [30–33]; however, that rate is still higher than that in the present study. Our results showed that some of the cases of PLO with vertebral fracture were treated as osteoporosis, although other types of premenopausal osteoporosis seldom received such treatment. In clinical practice, physicians often fail to consider fractures as low-energy, especially when dealing with young people, because osteoporosis is still perceived as a disease of elderly women. In addition, the availability of bone densitometry in each clinic can have affected the frequency of the tests.

The rate of medication use following PLO should also be further improved. The findings that half of PLO patients with vertebral fractures within 6 months after childbirth failed to get anti-osteoporosis drug therapy might indicate that even such fractures might be regarded as high-energy fractures in clinical practice. On the other hand, as a limitation of a database study, it cannot be ruled out that the fractures in the present study group were not actually low-trauma fractures. The low osteoporosis treatment rate might be due to high-energy trauma.

Meanwhile, it might be worrying that the physicians treating PLO in Japan preferred vitamin D or calcium supplementation therapy rather than a drug with stronger evidence for the prevention of fracture. Considering that patients with PLO are still in the child-bearing age, teriparatide should be prescribed in more cases than bisphosphonates. Furthermore, lactation inhibitors were seldom given, even though the lactation periods were unknown. Physicians might consider PLO to be a transient condition or lack understanding about the therapeutic effects of lactation cessation. Obstetricians should be able to recommend suppression of lactation to women who experience fragility fracture during pregnancy or just after childbirth considering its detrimental effect on bone mass. Kurabayashi et al. [10] reported that osteoporosis or osteopenia of the lumbar spine in the postpartum period could predict their persistence after 5–10 years. In addition, Kyvernitakis et al. [9] reported the subsequent fracture risk of patients with PLO after a median of 6 years of follow-up. These studies showed the importance of appropriate treatment and continuous follow-up for patients with PLO.

Our study also found many cases with premenopausal osteoporosis other than PLO (Table 2; Fig. 2). Although distal radius and sacral fractures were frequent among cases of premenopausal osteoporosis, neither was sufficiently treated as osteoporosis. We suspect that many women whom physicians considered as trauma patients may have underlying low BMD. A history of prior fracture at any site for all ages is considered an important risk factor for future fractures [19, 32, 34], particularly a fracture before menopause [1]. Paying greater attention to all cases of fragility fractures in young women might lead to more timely interventions that can reduce later fracture risk.

This study has some limitations that are mainly derived from its nature as a database study. First, PLO is a clinical diagnosis that requires that all causes of secondary osteoporosis and other conditions that may interfere with bone health have been ruled out. However, the database used does not provide data such as weight, height, medication history, lifestyle, smoking, or family history of osteoporosis. In addition, BMD values, the lactating periods, the resumption date of menstruation, and the menopause age of the participants were not known. If such clinical details were available, a more meaningful outcome might have been achieved. Some women with a code for “Absent, scanty, or rare menstruation” might have gone through menopause. Second, the number of parous women in the database might actually be greater. Because normal vaginal delivery is not covered by insurance in Japan, women who delivered normally could sometimes have lacked ICD-10 delivery codes. Therefore, we designed a complex process to definitely extract women who gave birth (Fig. 1). Even though women who delivered normally lacked ICD-10 delivery codes, they usually had procedure codes indicating vaginal delivery. In addition, the ledger data were helpful to extract normal delivery cases. However, the ledger data did not define the date of childbirth in cases where each parent participated in a different insurance scheme as employees, and the baby was covered under the same insurance scheme as the father. Thus, some potential cases might have been missed during the process of creating the study group. Third, without medical records, it cannot be entirely ruled out that the fractures in the study group were not actually caused by low-energy trauma, which might be associated with the low rate of prescriptions of drugs for the treatment of osteoporosis. In addition, the present study group consisted only of parous women. A study including nulliparas may lead to different outcomes for premenopausal osteoporosis.

Nevertheless, the main strength of the present study of premenopausal osteoporosis including PLO, a rare and often underdiagnosed condition, was the large sample size, since a large-scale nationwide claims database in Japan was used.

## Conclusions

PLO with vertebral fracture was one of the major types of premenopausal osteoporosis, as were distal radius fracture and sacral fracture in the periods away from childbirth. We epidemiologically demonstrated that pregnancy and lactation, a common life event for the majority of women, contribute to an increased risk of clinical fracture, especially among those with ovulation disorders, a history of infertility treatment, and high maternal age. The prevalence of PLO is considered to be higher than previously thought, probably owing to non-specific symptoms, indicating there might be a number of overlooked patients. It was also shown that PLO with vertebral fracture was mainly diagnosed a few months after childbirth and possibly up to 1 year later. Moreover, given the association of a hypoestrogenic condition and aging with the development of PLO, we presume that the temporal distribution of PLO might be related to not only high bone turnover due to lactation, but also the delayed resumption of ovarian function after childbirth. Most cases of premenopausal osteoporosis are overlooked and undertreated in Japan. Even those with PLO with vertebral fracture do not receive adequate treatment, despite such patients still undergoing more medical interventions than those with other types of premenopausal osteoporosis. The current trend of increasing maternal age in developed countries might be associated with an increase in the prevalence of PLO. More attention to and appropriate medical interventions for all types of fragility fractures in young women are recommended to help reduce the risk of fracture in the future. In addition, more timely interventions for PLO might lead to the improved management of latent patients with premenopausal osteoporosis.

## Data Availability

The datasets generated and/or analyzed during the current study are not publicly available because of ethical restrictions, but are available from the corresponding author or ST-M on reasonable request.

## Abbreviations

ASM: Antiseizure medication
ATC: Anatomical Therapeutic Chemical
BMD: Bone mineral density
DM: Diabetes mellitus
DPC: Diagnostic Procedure Combination
EphMRA: European Pharmaceutical Market Research Association
GC: Glucocorticoid
ICD-10: International Classification of Diseases, 10th revision
JMDC: Japan Medical Data Center
OR: Odds ratio
PLO: Pregnancy and lactation-associated osteoporosis
PRL: Prolactin
PTHrP: Parathyroid hormone-related protein
RA: Rheumatoid arthritis
SERM: Selective estrogen receptor modulator
WHO: World Health Organization
